# Personalized remotely guided preventive exercise therapy for a healthy heart (PRIORITY): protocol for an assessor-blinded, multicenter randomized controlled trial

**DOI:** 10.3389/fcvm.2023.1194693

**Published:** 2023-06-29

**Authors:** Camille De Wilde, Youri Bekhuis, Tatiana Kuznetsova, Jomme Claes, Guido Claessen, Karin Coninx, Elise Decorte, Delphine De Smedt, Dominique Hansen, Matthias Lannoo, Emeline M. Van Craenenbroeck, Nick Verhaeghe, Véronique A. Cornelissen

**Affiliations:** ^1^ Research Group of Rehabilitation of Internal Disorders, Department of Rehabilitation Sciences, Faculty of Movement and Rehabilitation Sciences, KU Leuven, Leuven, Belgium; ^2^ Department of Cardiovascular Sciences, Faculty of Medicine, KU Leuven, Leuven, Belgium; ^3^ REVAL—Rehabilitation Research Centre, Faculty of Rehabilitation Sciences, Hasselt University, Diepenbeek, Belgium; ^4^ Department of Cardiovascular Diseases, University Hospitals Leuven, Leuven, Belgium; ^5^ HCI And eHealth, Faculty of Sciences, Hasselt University, Diepenbeek, Belgium; ^6^ Department of Public Health and Primary Care, Interuniversity Centre for Health Economics Research, Ghent University, Ghent, Belgium; ^7^ Nutrition & Obesity Unit, Clinical and Experimental Endocrinology, Department of Chronic Diseases, Metabolism and Aging, KU Leuven, Leuven, Belgium; ^8^ Department of Cardiology, Antwerp University Hospital, Antwerp, Belgium; ^9^ Department of Medicine and Health Sciences, University of Antwerp, Antwerp, Belgium; ^10^ Department of Public Health, Interuniversity Centre for Health Economics Research, Vrije Universiteit Brussel, Brussels, Belgium; ^11^ Department of Cardiology, Heart Centre Hasselt, Jessa Hospital, Hasselt, Belgium; ^12^ Department of Public Health and Primary Care, Leuven Biostatistics and Statistical Bioinformatics Center (L-BioStat), KU Leuven, Leuven, Belgium; ^13^ Department of Cardiology, University Medical Center Utrecht, Utrecht, Netherlands

**Keywords:** exercise, personalized, heart failure, prevention, obesity, diabetes, cost-effectiveness (economics), randomized controlled trial

## Abstract

**Aims:**

A key treatment for patients with varying stages of heart failure with preserved ejection fraction (HFpEF) is exercise. Yet, despite a Class 1A recommendation, only one-third of patients exercise sufficiently. A huge treatment gap exists between guidelines and clinical practice. PRIORITY aims to establish the feasibility, clinical effectiveness and cost-effectiveness of a hybrid centre and home-based personalized exercise and physical activity intervention for patients along the HFpEF continuum.

**Methods:**

An assessor-blinded, multicenter randomized controlled trial will be conducted among 312 patients along the HFpEF continuum. Participants will be randomized (1:1) to the PRIORITY intervention or a comparator group receiving only a written exercise prescription. Participants in the PRIORITY group will receive 18 supervised centre-based exercise sessions during one year, supplemented with a remotely guided home-based physical activity program. Outcomes will be assessed at baseline, 4 months, one and two years. The primary outcome is the peak oxygen uptake (pVO2) at 1-year. Secondary outcomes include physical activity, other physical fitness parameters, cardiovascular health, echocardiographic parameters, health-related quality of life and costs at 1-year FU. Machine learning algorithms will analyse big data on physical activity collected during the 1-year intervention to develop models that can predict physical activity uptake and adherence as well as changes in fitness and health. A cost-utility analysis will be performed to evaluate the cost-effectiveness of the PRIORITY intervention compared to the control condition.

**Discussion:**

We anticipate that participants in the supervised home-based exercise intervention group will have a greater increase in pVO2 compared to those receiving a written exercise prescription.

**Trial registration number:**

This trial is registered at ClinicalTrials.gov (NCT04745013) and is currently in the recruitment stage.

## Background

Heart failure (HF) is a rapidly growing health problem with an estimated prevalence of 64.3 million people worldwide, which poses a major burden on public health and healthcare (1) Approximately half of the HF population has a preserved ejection fraction (HFpEF, left ventricular ejection fraction ≥50% (2). This syndrome is promoted by cardiovascular (CV) risk factors (stage A) such as obesity, exercise deficiency, hypertension and diabetes (2, 3). With increasing age these risk factors frequently result into structural and functional heart alterations without (stage B) or with (stage C) HF signs or symptoms such as exertional breathlessness, exercise intolerance and muscle fatigue (3, 4). With the extending life expectancy and increasing prevalence of CV risk factors, the prevalence of HFpEF as well as its burden on societal health are expected to increase over the next decades (5).

Thus, early and long-term preventive strategies are urgently needed to improve health-related quality of life and prognosis of patients along the continuum of HFpEF. Physical activity and exercise are recognized as effective interventions to prevent premature CV mortality and CV disease progression (6, 7). Therefore, exercise training gained a Class IA recommendation in cardiovascular disease (CVD) management and prevention, not only in patients but in all adults, especially in those with CV risk factors (8–10). How to provide and make it accessible to all? Given the rapidly expanding group of patients at risk for developing symptomatic HF in modern society, centre-based exercise programs are unlikely to gain acceptance as a cost-effective means of preventing progression towards overt HFpEF. Moreover, in current daily practice, participation and compliance rates are notoriously poor (11–13). The incorporation of exercise in an early comprehensive long-term care plan remains largely neglected and severely underused as confirmed by the European EUROASPIRE surveys (12). Also, the beneficial effect of a centre-based exercise intervention is often attenuated when the exercise intervention stops and the patient relapses into a sedentary lifestyle. As such, there is a critical need to facilitate the accessibility and affordability of structured, personalized exercise interventions in the home environment in order to increase uptake, effectiveness, and long-term adherence to exercise training in CV disease.

In this regard, home-based exercise may also be more promising from an health-economic point of view. To our knowledge, no studies yet examined the clinical and cost-effectiveness of remotely guided home-based exercise therapy in the prevention of overt HFpEF.

## Aims and objectives

The PRIORITY trial will evaluate the feasibility, clinical and cost-effectiveness of a hybrid (centre and home-based) personalized exercise and physical activity intervention to prevent the deleterious effects of sedentary ageing on the heart and forestall the development and progression towards overt HFpEF.

### The primary objective of the trial

The primary objective of the PRIORITY trial is to compare the effects of a hybrid personalized exercise intervention against an exercise prescription only in improving the peak exercise capacity (pVO_2_) of patients along the continuum of HFpEF. We hypothesize that the effect on pVO_2_ will be significantly larger in the experimental group (PRIORITY group) compared to the comparator group (written exercise prescription only) at one year.

### Secondary objectives of the trial

• To compare daily step count of patients at one year (key secondary outcome) and two years of follow-up (FU)
• To compare weekly minutes of moderate to vigorous physical activity at one year (key secondary outcome) and two years of FU
• To assess the safety of the PRIORITY intervention
• To compare pVO_2_ at 4 months and two years of FU
• To compare health-related fitness components (body composition, submaximal exercise capacity, muscle strength and muscle endurance) at 4 months, 1-year and 2 years of FU
• To compare traditional CV risk factors at 1 year and 2-year of FU
• To compare changes in echocardiographic parameters of left ventricular systolic and diastolic function at rest and during exercise at one year of FU
• To compare changes in neurohumoral activation (NT-proBNP) at one year of FU
• To compare psychosocial well-being and health-related quality of life at 1-year and 2-year of FU
• To assess the implementation potential of the PRIORITY intervention in the Flemish healthcare setting
• To evaluate the cost-effectiveness of the PRIORITY intervention compared to the control condition
• To facilitate the creation of more personalized interventions and better-tailored exercise prescriptions to maximize their therapeutic effect by developing machine learning models which predict uptake of physical activity behaviour and changes in physical activity and cardiorespiratory fitness.

## Methods

The PRIORITY trial protocol is written following recommendations from the Standard Protocol Items: recommendations for interventional trials (SPIRIT) checklist (14). This trial has been prospectively registered at ClinicalTrials.gov: (NCT04745013) on the 2nd of April 2021. Ethical approval has been obtained from the Ethics Committee of UZ/KU Leuven; Ethics Committee of UZA; Ethics Committee of Jessa Hospital and Ethics Committee of UHasselt.

### Trial design

**Figure 1:**
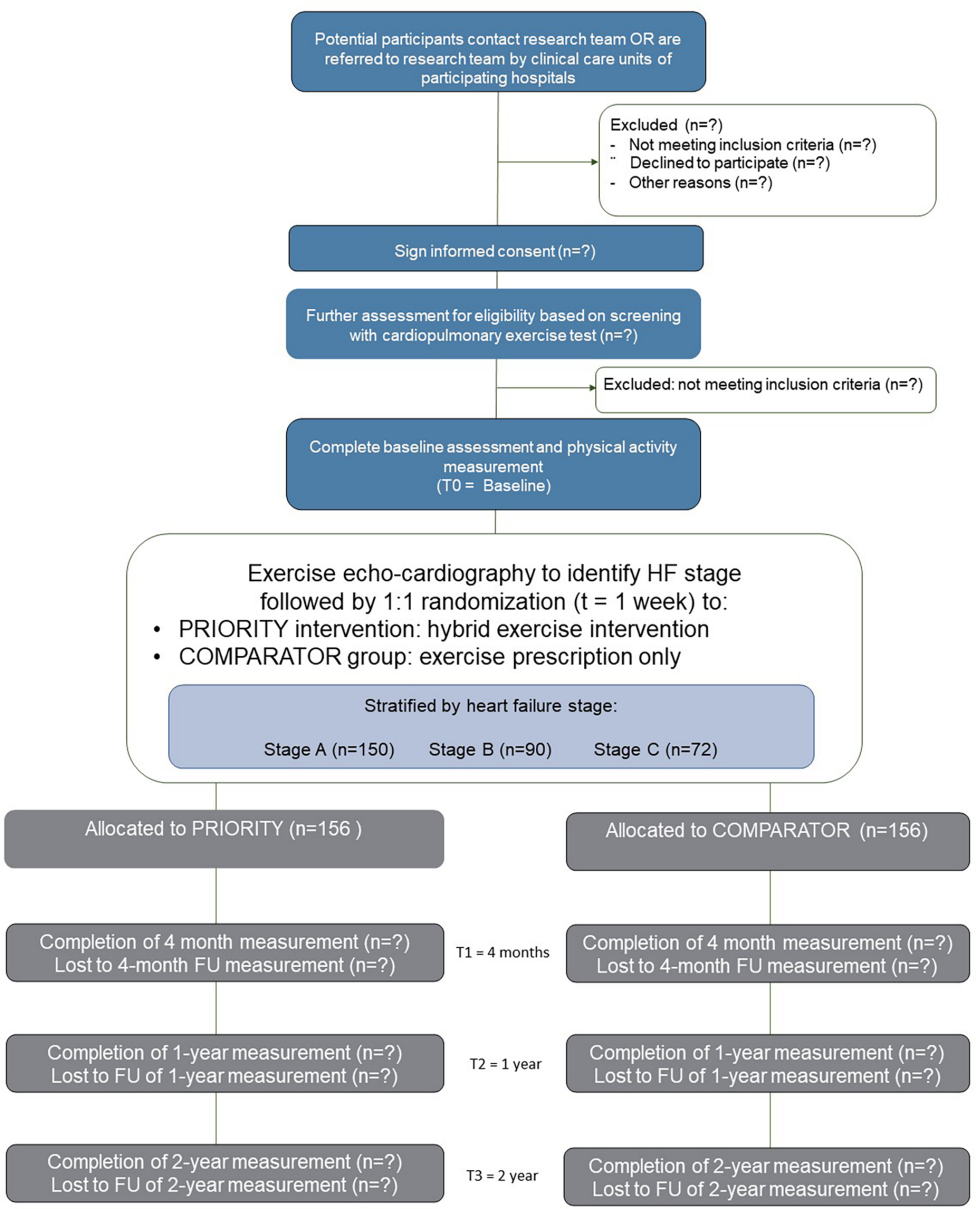
Trial flow.

The PRIORITY trial is designed as a 2-year prospective randomized, controlled, assessor-blinded multicenter comparative trial with two parallel groups. The research approach has similarities to a hybrid type I effectiveness-implementation research design (15). The primary focus is to evaluate the effectiveness of a hybrid exercise and physical activity intervention, whilst concurrently gathering information on its potential for implementation in a real-world setting. Researchers performing the outcome analyses will be blinded to group allocation. Figure 1 shows the trial flow.

### Trial setting

The PRIORITY trial will be conducted at three sites in Belgium: University Hospital Leuven—University of Leuven, Antwerp University Hospital, and Jessa Hospital Hasselt—Hasselt University. Recruitment will be performed via advertisements, flyers and social media and the different clinical units (e.g., hypertension clinic, obesity clinic and HF clinic) of the hospitals. In addition, eligible participants of the FLEMENGHO cohort (https://flemengho.eu/en/) visiting the University Hospital Leuven will be invited for participation. The University of Leuven will be the coordinating centre for the trial. Daily management of the trial will be performed by a local principal investigator (VC, DH, EVC) at each participating site.

### Eligibility criteria and recruitment

The trial population will comprise 312 men and women (aged ≥30 years) along the continuum of HFpEF who are on optimal medical treatment and stable regarding symptoms and pharmacotherapy for at least 4 weeks before enrolment in the trial. All patients should have internet access at home. To represent the distribution of HFpEF stage A–C in the population, this will include 150 patients with HFpEF stage A, 90 patients with HFpEF stage B and 72 patients with HFpEF stage C. Participants will be recruited during a 2-year period which started in September 2021. Local investigators at each participating site will assess patient eligibility. A detailed description of the inclusion and exclusion criteria is shown in Table 1. Potentially eligible patients will then be contacted by a member of the research team and provided with a full oral explanation of the design and purpose of the trial, responsibilities of the participants, reasonably foreseeable inconveniences, and confidentiality of the information collected.

**Table 1:** Inclusion and exclusion criteria for the PRIORITY trial.

HFpEF stage	Inclusion criteria	Exclusion criteria
A “at risk”	History of treated or untreated hypertension (blood pressure 130/90–159/99 mmHg) **AND/OR** Prediabetes with either: • Fasting plasma glucose: 100–125 mg/dl (5.6–6.9 mmol/L) • HbA1c: 5.7%–6.4% • HOMA-IR >2.0 **AND/OR** Obesity: 30 kg/m² ≥ BMI ≤ 42 kg/m² **AND/OR** • Diabetes treated with anti-diabetics	• Significant illness during the last 6 weeks • Known severe arrhythmia (i.e., the occurrence of complex ventricular arrhythmia or other arrhythmia, including second- or third degree AV block, that interferes with normal maintenance of cardiac output during exercise and with functional or prognostic significance) • Significant myocardial ischemia (defined as marked ST displacement (horizontal or downsloping of >2 mm, measured 60–80 ms after the J point at the end of the QRS complex) or increasing chest pain, hemodynamic deterioration, or exercise-induced arrhythmia at baseline testing • Co-morbidity that may significantly negatively influence 1-year prognosis (e.g., oncological diseases, auto-immune disorders, etc.) • Non-cardiac causes for heart failure-like symptoms ◦ Severe chronic obstructive pulmonary disease (FEV1 <50%) ◦ NYHA class IV ◦ Significant peripheral artery disease (Fontaine > IIb) ◦ Specific cardiomyopathy (e.g., amyloidosis, congenital heart disease) ◦ Chronic kidney disease (eGFR < 30 ml/min) or on dialysis • Participation in another clinical intervention trial • Any inability or contraindication to perform a cardiopulmonary exercise test or to participate in an exercise program (physiological, physical, or mental) as considered by the supervising physician (KG, GC, EVC) • Inability to fill in questionnaires presented in Dutch. • Advanced HF (stage D)
B “pre-HFpEF”	CV risk factors as mentioned under stage A **AND** Subclinical signs of left ventricular diastolic dysfunction and/or raised left ventricular filling pressures (see Table 2.)	
C “symptomatic HF”	Criteria as mentioned under stage B with either: • Heart failure hospitalisation **AND/OR** • Treatment with diuretics **AND/OR** • Symptoms and/or signs compatible with a diagnosis of heart failure with an HFA-PEFF total score of ≥5	

HbA1c, hemoglobin A1c; HOMA-IR, homeostatic model assessment of insulin resistance; BMI, body mass index; CV, cardiovascular; HFA-PEFF, heart failure association—pretest assessment, echocardiographic and natriuretic peptide score, functional testing in case of uncertainty, final etiology.

### Randomization and concealed allocation

Upon written informed consent and following the baseline measurements including a fasting blood draw, cardiopulmonary exercise test (CPET) and a rest and exercise echocardiography, patients will be randomized to either the PRIORITY group or the comparator group with a 1:1 allocation ratio stratified by HFpEF stage, as shown in Table 1. Asymptomatic patients with HF risk factors such as hypertension, (pre)diabetes, and obesity but free from echocardiographic abnormalities will be classified as stage A. Early HF phenotypes (stage B) will be defined as asymptomatic patients with HF risk factors and echocardiographic evidence of cardiac structural and functional abnormalities, consistent with the presence of left ventricular diastolic dysfunction and/or raised left ventricular filling pressures as defined by 2 out of 4 ESC criteria (Table 2) (16). Patients with symptomatic HF (stage C) will be characterized as symptomatic patients with an HFA-PEFF score of ≥5 (4). For patients with an intermediate HFA-PEFF score after resting echocardiography, the results of the diastolic stress test will be included in the score [i.e., average *E*/*e*′ ≥15 adds 2 points and an average *E*/*e*′ ratio ≥15 with a peak tricuspid regurgitation (TR) velocity >3.4 m/s adds 3 points to the previous score after the resting echocardiography]. Randomization schedules are generated using a computerized random number generator. Randomization is being performed by an independent designated member of the coordinating centre (KU Leuven) in response to a request from a local investigator, thus assuring concealed allocation and minimizing selection bias.

**Table 2:** Objective evidence of cardiac structural or functional abnormalities consistent with the presence of left ventricular diastolic dysfunction/increased left ventricular filling pressures.

Parameter	Threshold
LV mass index (LVMI)	≥95 g/m^2^ (♀), ≥115 g/m^2^ (♂)
or Relative wall thickness (RWT)	>0.42
LA volume index (LAVI)	>34 ml/m^2^ (SR), >40 ml/m^2^ (SR)
Average (septal and lateral) *E*/*e*′ ratio at rest	>9
Estimated PA systolic pressure	>35 mmHg
TR velocity at rest	>2.8 m/s

### Sample size calculation

The power calculation is based on a constrained longitudinal data analysis (cLDA) model (17, 18) and calculated using an approach presented by Stroup (19). To have at least 90% power to detect a difference of 2.7 ml/kg/min in pVO2 after 12 months, 90 subjects per group (180 in total) are required based on a two-sided test and setting alpha equal to 0.05. The effect size of 2.7 is a weighted average of the expected effects in the three stages i.e., 0.20 × 1 ml/kg/min (Stage C) (20, 21) +0.30 × 2.5 ml/kg/min (Stage B) +0.50 × 3.5 ml/kg/min (Stage A) (22, 23). A common standard deviation of 5, a correlation between baseline and 12 months equal to 0.50 and a drop-out rate at 12 months as high as 40% were assumed (24). However, the sample size will be increased to 312 patients in total (156 per group) such that for two key secondary outcomes standardized effect sizes as small as 0.45 and 0.40 can be detected with more than 90% and 80% power, respectively (using a cLDA model assuming—as for the primary outcome—a baseline-year correlation of 0.50% and 40% dropout at 12 months). For example, for the key secondary outcome (moderate to vigorous physical activity, MVPA) a standardized mean difference equal to 0.42 corresponds to a difference of 1,235 steps (25). Note that for the power calculation of the key-secondary outcomes an alpha-level of 0.05/2 = 0.025 was used.

### Trial intervention

#### Experimental group: PRIORITY intervention

**Figure 2:**
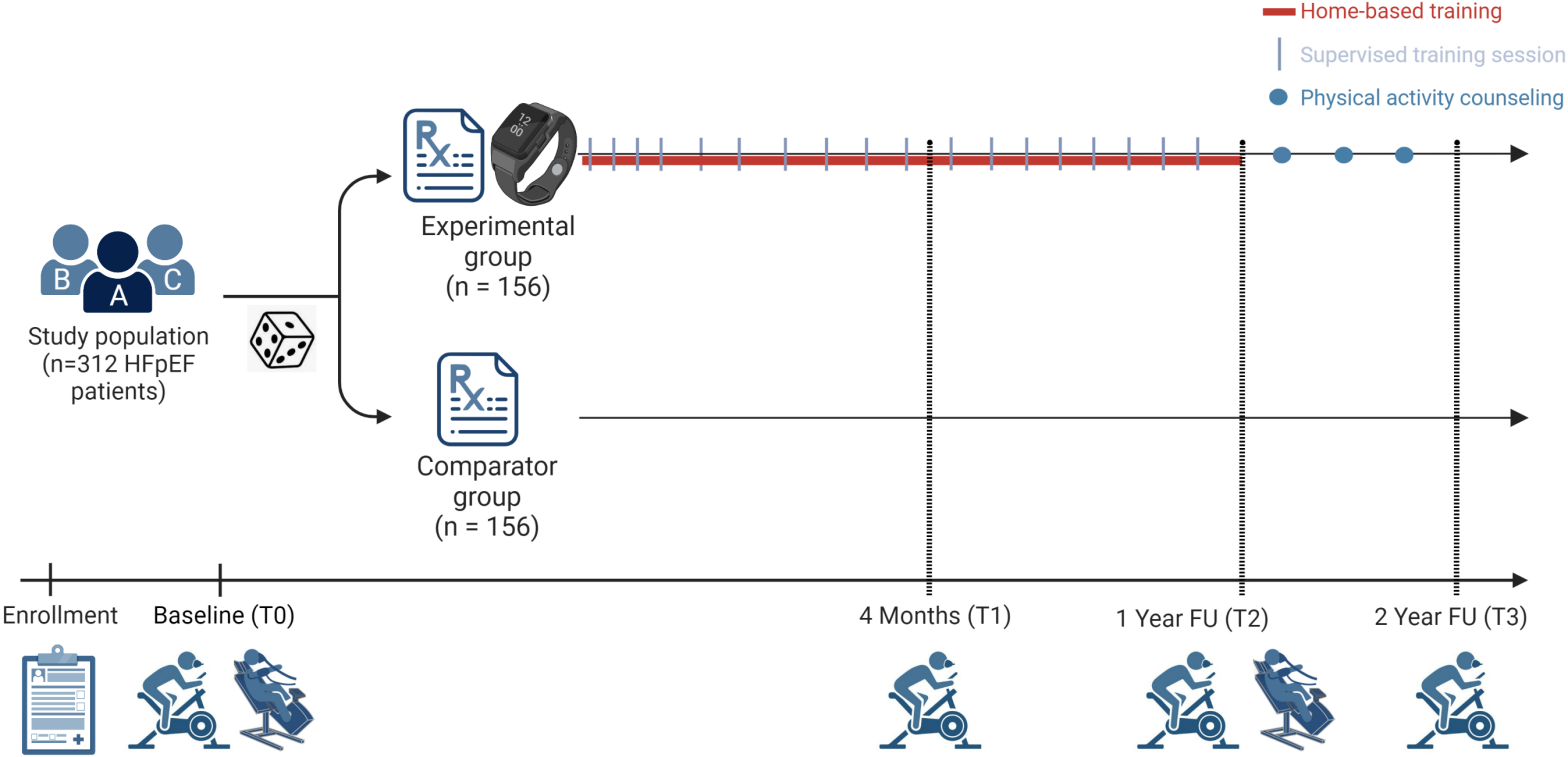
Intervention of the PRIORITY trial.

Patients in the PRIORITY group will participate in a 1-year hybrid exercise and physical activity intervention, consisting of 18 supervised exercise sessions and a remotely monitored home-based physical activity program. Following a CPET, patients randomized to PRIORITY will receive a personalized exercise prescription generated by the EXPERT tool (26), a Garmin sports watch and chest strap (Garmin Forerunner 45, Garmin Ltd. Kansas, USA) and access to a web-based exercise platform (www.inspanningstherapie.be). Patients with HFpEF stage C will also receive a home ergometer. Subsequently, the automatically generated exercise prescription will be fully person-tailored by one of the physiotherapists (CD, SN) or a physical therapist (ED) of the research team following the FITT-VP (frequency, intensity, time, type, volume, and progression) principle during the first one-on-one consultation and considering patients’ needs, barriers and goals. Over a period of one year, with gradually increasing time intervals between sessions, patients will be invited to participate in 18 supervised centre-based exercise sessions, using the checklist for intervention description and replication (TIDieR) (27) as described in more detail in Supplementary File S1. This number of sessions was chosen as this is the number of sessions patients get reimbursed for other injuries (e.g., shoulder pain, muscle injuries, etc.) when consulting a physiotherapist in Belgium. While providing supervised exercise, patients are acquainted with the use of the sports watch, the exercise platform, heart rate training zones and intensity levels, strength, and balance exercises. Additionally, facilitators and barriers will be discussed, including issues they encounter when (trying to be) being physically active at home. In this way adaptations to the exercise program could be made and follow-up will be regularly provided. Patients will be asked to register each exercise session (supervised and home-based) with sports watches and to upload their training data (including heart rate data, steps, distance travelled, floors climbed, speed, …) to the Garmin Connect software (Garmin Ltd. Kansas, USA). In addition, they will be requested to subjectively rate the intensity of the session using the BORG scale. Training sessions focus on aerobic endurance training and dynamic strength training and also include balance, coordination and flexibility when needed. As depicted in Figure 2, the number of supervised sessions gradually decreases over time to maximally encourage self-management and empowerment: month 1 (one weekly supervised session)—month 2–4 (one supervised session every 2 weeks)—month 5–12 (one supervised session per month). To enhance adherence to the intervention, patients will be contacted by the physiotherapist (via e-mail or phone according to preference) if six consecutive prescribed home-based sessions are missed.

#### Comparator group

Patients randomized to the comparator group will be advised to be physically active and will receive a personalized exercise prescription as automatically generated by the EXPERT tool and orally explained to the patient (26). Subsequently, patients are free to participate in any form of physical activity or structured exercise. However, there will be no contact between the patients and the investigators to provide feedback or support during the 1-year FU period, except for the four-month FU assessment.

#### Concomitant care

All patients will continue to receive usual care which includes optimal medical and pharmacological treatment during the trial.

##### From year one to year two

As shown in Figure 2, all patients will receive an overview of the change of their physical fitness test results one year after their baseline measurements. Patients in the PRIORITY group will then be invited for four physical activity consultations: immediately at month 12 and then subsequently at month 15, month 18 and month 21 to discuss their current physical activity behaviour and further co-design their physical activity program addressing barriers and enablers of physical activity. The key component of the physical activity intervention consists of motivational interviewing to evoke intrinsic motivation to support long-term behavioural change. Patients of the PRIORITY group will still be able to make use of the web-based exercise platform, their resistance training bands, their sports watch and chest strap. Patients with HFpEF stage C will also keep their home-ergometer. Patients in the comparator group will not be contacted during this year.

##### Monitoring and promoting adherence during a 2-year period

Strategies to improve adherence to an active lifestyle include a home-based mode of training, gradually decreasing in-person follow-up sessions, user-friendly training software (training diary + prescription with pre-recorded videos and pictures), periodized training volume, and personalized training intensities (relative training intensities based on CPET), co-development of an activity intervention adapted to the patient’s preferences and by the use of motivational interviewing techniques, SMART goal setting, self-monitoring of physical activity behaviour and gradually decreasing follow-up prompts.

#### Outcome measures and data collection

Assessments will be performed at baseline before randomization (T0), at 4 months (T1) as this is the median intervention duration in most supervised exercise studies, at the end of the 1-year intervention (T2) and after two years of FU (T3). The main goal is to assess the effectiveness of the intervention on different biopsychosocial outcomes at 1- and 2-year FU. A tabulated overview of the primary, secondary and other outcomes measured at the different time points is provided in Table 3.

**Table 3:** Tabulated summary of trial schedule.

Outcomes	Instrument	T0	T1	T2	T3
Primary outcome					
Exercise capacity	Peak oxygen uptake via cardiopulmonary exercise test on cycle ergometer	X	X	X	X
Secondary outcomes					
Physical activity	ActiGraph GT9X Link	X	X	X	X
Health related physical fitness					
Body morphology/composition	Body mass (body weight scale), height (stadiometer)and waist circumference (stretch-resistant measuring tape). Bioelectrical impedance will be used to assess fat and fat-free mass.	X	X	X	X
Handgrip strength	Isometric handgrip grip strength of both hands using a Jamar Hydraulic Hand Dynamometer (Sammons Preston Inc.).	X	X	X	X
Quadriceps muscle strength	Three voluntary maximal isometric contractions (6 s) performed at a 60° angle of the knee, with a 60-second rest period between each test, on a dynamometer (Biodex Medical Systems Inc., 840–000 System 4, New York, USA).	X	X	X	X
Quadriceps muscle endurance	25 repetitive maximal isokinetic knee extensions at 180°/s, performed on a dynamometer. Mean torque and percentage decrement score will be used as markers for muscle endurance.	X	X	X	X
Cardiovascular health					
Blood sampling	Glucose homeostasis, blood lipid profile, NT-proBNP	X		X	X
Rest and stress echocardiography	Vivid E95 ultrasound scanner, semi-supine bicycle ergometer (Ergoline) is used for stress echocardiography.	X		X	X
Health related QoL & psychosocial wellbeing	EQ-5D-5l Questionnaire	X	X	X	X
	SF-36 Questionnaire	X	X	X	X
	Exercise Self-Efficacy Scale	X	X	X	X
	Exercise Barriers Questionnaire	X	X	X	X
	Social Support Questionnaire	X		X	X
Implementation potential	User experience Questionnaire			X	** **
	Adherence to exercise program			X	** **
	Patient Debriefs: Self-reported and objective measures (exercise log, heart rate measurements)			X	** **
Evaluation towards cost-effectiveness	Productivity Cost Questionnaire	X	X	X	X
	Medical Consumption Questionnaire	X	X	X	X
	*Quality of life* via *EQ-5D-5l questionnaire*	X	X	X	X
Safety monitoring	Adverse events reporting by patients	X——————––X			
Other outcomes					
Sociodemographic characteristics	General Questionnaire	X	X	X	X
Substudy: muscle metabolism	Device for muscle oxygenation measurements (PortaMon)	X		X	
Substudy: vasoreactivity/vasomotor function	Flow mediated slowing via Vicorder	X		X	

T0, baseline; T1, measurement at 4 months; T2, measurements at 1 year; T3 measurements at 2 years; NIRS, near infrared spectroscopy; FMS, flow mediated slowing.

#### Primary outcome measure

Our primary outcome will be the cardiorespiratory fitness expressed as pVO2 during a graded maximal CPET on a bicycle until exhaustion (JAEGER Vyntus CPX, Vyaire medical, USA). pVO2 is determined as the highest attained peak VO2 during an average of 30 s of exercise. We choose pVO2 as our primary outcome because it has been shown to be the most important independent predictor of CV morbidity and mortality in patients with HFpEF stage A–C (28). A 5 + 5 W/min, 10 + 10 W/min, 20 + 20 W/min, or 50 + 25 W/min continuous ramp protocol will be used according to the participants’ estimated fitness level to ensure a CPET duration between the recommended 8–12 min (29). A 12-lead electrocardiogram will be monitored continuously, and gas exchange will be measured breath-by-breath. Blood pressure will be measured automatically every 2 min (SunTech Tango M2, SunTech Medical, USA). After reaching maximal exertion, the patients will cycle another 3 min to measure recovery heart rate, blood pressure, rating of perceived exertion and to document the reason for test termination. All raw CPET data will be forwarded for analysis to a blinded CPET core lab at KU Leuven to ensure reliable analysis of the data.

#### Secondary outcome measures

##### Physical activity

A validated tri-axial accelerometer (ActiGraph™ GT9X Link, ActiGraph LLC, Pensacola, Florida, USA) will be used to objectively measure the patient’s physical activity level (30). Participants will be asked to wear the ActiGraph GT9X Link on the non-dominant wrist for 24 h/day for 7 days. Measurements will be considered valid when at least 3 weekdays and 1 weekend-day of 10 h wear-time have been recorded (31). ActiLife software will be used to extract the raw data from the physical activity monitor which will then be transferred to the physical activity core lab at KU Leuven for offline analysis. The following parameters will be determined: number of steps (key secondary outcome), energy expenditure (total and active), time spent doing MVPA (key secondary outcome) and number of sedentary bouts.

##### Muscular fitness

A maximal voluntary isometric handgrip strength test will be performed using a JAMAR dynamometer (JLW Instruments, Chicago, Illinois, USA) (32). For each hand, the patient will perform three repetitions while sitting upright on a chair and with the elbow at 90° flexion. The maximum value (in kg) for each hand will be recorded. Additionally, muscle strength and endurance of the quadriceps muscle will be tested in the right leg using a Biodex system 3 Pro (Biodex Medical Systems Inc., Shirley, New York, USA) (33). Patients will be instructed to perform three voluntary maximal isometric quadriceps contractions for 6 s, interspersed with a 1 min rest period. The highest value will be used in the analysis as a measure of isometric power. This will be followed by two bouts of 25 repetitive maximal isokinetic knee extension-flexion movements interspersed with a 2 min recovery period.

##### Body Composition

Body composition will be measured in the supine position using Bodystat 1,500 (Bodystat Ltd, Douglas, Isle of Man, UK) (28). Anthropometric characteristics such as length (stadiometer) and body mass will be measured to the nearest 0.1 cm and 0.1 kg in fasting state and light clothing to calculate body mass index (kg/m²). Waist circumference is measured using non-elastic tape.

##### Blood biochemistry

A blood sample will be drawn with the patient in a fasting state and biochemical analysis includes fasting glucose, hemoglobin A1c, total cholesterol, low-density lipoprotein cholesterol (LDL-C), high-density lipoprotein cholesterol (HDL-C), triglycerides, creatinine and NT-proBNP.

##### Rest transthoracic echocardiography

Comprehensive two- and three-dimensional echocardiography will be performed by experienced sonographers using a standardized protocol (see Supplementary File S2). All recordings will include at least 3 cardiac cycles and qualified readers will interpret the images offline using EchoPAC software (version 204, GE Vingmed, Horten, Norway). The standard protocol will include conventional cardiac dimensions, mass, and systolic and diastolic function in accordance with contemporary international guidelines (34, 35).

*Exercise echocardiography combined with CPET (CPET-echo)* will be performed following a standardized protocol as described before (see Supplementary File S2) (36–40). Exercise will be performed on a semi-supine bicycle ergometer (Ergoline GmbH, Bitz, Germany) with a continuous ramp protocol aiming for a total exercise duration of 10–12 min (60–65 rotations/min). Images will be acquired at rest, low intensity (HR between 90 and 100 beats per min, before fusion of E and A waves, or at an RER between 0.85 and 0.9 when chronotropic incompetence is present), and at peak exercise (RER >1.05). Loop registration of at least 10 beats will be made to overcome the expected decrease in acoustic quality caused by hyperventilation. At one year of follow-up, the CPET-echo will follow the same imaging protocol. The power output at low-intensity exercise will be identical to the low-intensity workload during the baseline CPET-echo at the time of inclusion. In contrast, the power output of the peak exercise stage will be determined based on the criterion of achieving RER 1.05. All analysis will be performed offline at the core lab of Leuven using EchoPAC software (version 204, GE Vingmed) in accordance with contemporary international guidelines (34, 35).

*Health-related quality of life (HRQoL)* will be assessed via the generic RAND-36 Questionnaire (SF-36), administered as an online survey via the web-based application REDCap (41). Here, different components are addressed: physical functioning (10 items), limitation due to physical health (4 items) or emotional problems (3 items), energy and fatigue (4 items), emotional well-being (5 items), social functioning (2 items), pain (2 items), general health (5 items) and perceived change in general health (1 item). All items are scored on a nominal or ordinal scale and transformed to a percentage of impairment (0% complete impairment, 100% no impairment). In addition, the EQ-5D-5l questionnaire will be administered covering five dimensions: mobility, self-care, usual activities, pain/discomfort and anxiety/depression. The health status profile can be translated into a utility value between 0 and 1, serving as input for the health economic evaluation.

##### Safety

Adverse events will be monitored for 2 years after randomization. All adverse events will be recorded and reported in accordance with the Good Clinical Practice decision tree for adverse event reporting and will be reported to the central research ethics committee. Serious adverse events (SAE) are defined as all-cause mortality, hospitalization for CVD or serious atrial or ventricular arrhythmia. Other adverse events will include training-related adverse events such as muscle, tendon or joint problems that will preclude exercise participation or other diseases that require an interruption of the exercise intervention.

*Implementation potential* will be evaluated by measuring adherence [defined as a % of recommended duration of exercise (minutes)] and compliance [will be calculated as % of time at the recommended intensity (i.e., within correct heart rate zone)] to the exercise intervention. These data will be collected using the GARMIN sports watch or exercise diary for those patients encountering difficulties using the watch. In addition, the usability and feasibility of PRIORITY will further be assessed using the Users Experience Questionnaire (UEQ) and the System Usability Scale (SUS) (42).

#### Other exploratory outcome measures

In a subsample, continuous wave *near-infrared spectroscopy* (NIRS, PortaMon, Artinis Medical systems, Elst, The Netherlands) measurements will be performed during the CPET to evaluate muscle oxygen saturation. NIRS relies on the light absorption properties of chromophores in the tissue of interest (e.g., hemoglobin and myoglobin in muscle). As the underlying myoglobin concentration tends to remain constant during exercise, the changes in the oxy- or deoxygenation signals can be attributed to changes in hemoglobin content (43, 44). In the same sample, *brachial artery vasoreactivity* will be evaluated by means of flow-mediated slowing (FMS), a technique that measures brachial pulse wave velocity deceleration to reactive hyperaemia via the brachial-radial oscillometric technique (Vicorder device, SMT medical technology GmbH, Würzburg, Germany) (45).

### Data management

Patient data will be anonymized using a personal identification code (PIC) on all case report forms and in the electronic database. Data will be recorded in hard copy at the time of the measurement and will subsequently be entered electronically in “REDCap (https://www.project-redcap.org)”, an open-source clinical trial software for electronic data capture and data management hosted at KU Leuven servers. Hard copies will be stored in a secured filing cabinet at the participating sites. Digital scans will be uploaded in the REDCap database and on the KU Leuven secure server. The type of activity that an individual user may undertake in the REDCap database is regulated by the privileges associated with his/her login credentials. Source data, randomization and pseudonymization lists are stored on a secure server of KU Leuven with password protection. All data analyses and reporting will be performed according to best practice and reported in agreement with Consolidated Standards of Reporting Trials guidelines (46). An independent researcher will regularly audit a randomly chosen subset of patients at each site to ensure adherence to the intervention program and the trial protocol. Any issues pertaining to nonadherence with the eligibility of a randomized participant, allocation of interventions, or concerns relating to adverse events are being discussed with and reviewed by the steering committee.

### Statistical analysis

The full analysis set (FAS) will, in accordance with the intent-to-treat principle, include all randomised patients according to their randomised treatment. The FAS will be used for the evaluation of all efficacy and safety endpoints. Patients from the FAS with major protocol deviations will be excluded from the per protocol set (PPS). A cLDA (18) model will be used to compare the pVO2 at 12 months between both groups. In this model, both the baseline pVO2 and post-baseline pVO2 values (4 months and at 12 months) are modelled as dependent variables, as opposed to a longitudinal ANCOVA model in which the baseline value is included as a covariate. The comparison at 12 months will be based on a two-sided test, setting alpha equal to 0.05. An unstructured covariance matrix will be used for the three longitudinal measurements. The HFpEF stage will be added as a factor in the model and the differences between the groups are allowed to differ between the stadia. The intervention effect will be calculated as a weighted average of the stage-specific effects. The estimation of the model will be likelihood based and results are therefore valid under the MAR (missing at random) assumption, i.e., subjects with a missing pVO2 value at a specific timepoint are assumed to be well represented by other subjects not having a missing value at that timepoint and having the same observed values at the other timepoints. Note that the comparison at 4 months, which will be derived from the same model, is a secondary outcome. The cLDA model (restricted to baseline and 12 months) will also be used for the comparison of the two key secondary outcomes, applying a Bonferroni-Holm correction.

#### Health economic evaluation

*The health economic evaluation* will evaluate health outcomes [expressed as quality-adjusted life years (QALY)] and costs of the PRIORITY intervention compared to usual care. A cost-utility analysis will be performed applying a decision-analytic Markov model to predict costs and health effects of the intervention vs. usual care. A lifetime time horizon will be considered. The analysis will be conducted from a societal perspective, meaning that both direct and indirect costs will be included. Direct costs will include direct medical costs (e.g., hospitalization, nursing care, medication) and direct non-medical costs (e.g., travel costs). Indirect costs are those related to lost human productivity (for example productivity losses due to morbidity or mortality). The cost of delivering the program will be collected alongside the RCT. Information on participants’ health care resource use and absence from work will be obtained by a researcher using a questionnaire at T0, T1, T2 and T3 as shown in Table 3. Subsequently, resource use unit costs will be attached to the above mentioned data using publicly available databases (47). The ratio of the incremental costs to the incremental health effects, i.e., the incremental cost-effectiveness ratio (ICER) will be calculated. The model will link changes in exercise capacity and cardiovascular risk profiles to recurrent cardiovascular events and CVD mortality to estimate the cost-per-QALY associated with the PRIORITY intervention compared to usual care (48). One-way sensitivity and probabilistic sensitivity analyses will be performed to address the uncertainty related to key input parameters of the Markov model. In the one-way sensitivity analysis, the impact of key input parameters (e.g., costs, transition probabilities) on the ICER will be examined by varying their values separately. A probabilistic sensitivity analysis, based on 5,000 iterations, will be performed to evaluate the uncertainty for key input parameters by varying them concurrently.

## Discussion

According to the latest WHO data, about 80% of CV disease is potentially avoidable with better management of CV risk factors such as diet and lifestyle. Therefore, the existing evidence advocates for a more aggressive preventive strategy including exercise and physical activity. Also, in patients with (risk factors for) HFpEF, there is no doubt that exercise is an imperative therapy in primary and secondary prevention (8, 16). Though, the implementation and accessibility of guidelines in daily clinical practice remains a challenge. Therefore, we initiated one of the largest multi-centre RCT that aims to validate the clinical impact and cost-effectiveness of a hybrid strategy to deliver person-tailored exercise therapy to patients along the HFpEF continuum.

The first strength of this approach is that it starts from a person-tailored exercise prescription using the EXPERT-tool and combines regular in person interaction with the support of technology. This allows us to move away from a healthcare provider-centric system and to empower patients in self-managing their health which is expected to increase adherence and compliance to the exercise and physical activity program. PRIORITY achieves this goal by bringing structured personalized exercise therapy to the patient’s home, which considers patient’s goals, co-morbidities, barriers, and enablers to exercise. Compared to the current situation, PRIORITY is designed with the aim to result in (1) a higher uptake of exercise therapy and physical activity by increasing accessibility (2) a better-sustained adherence to an optimally dosed exercise therapy and (3) a better clinical effectiveness of exercise therapy by providing a 1-year intervention.

Second, the PRIORITY trial applies an assessor-blinded, multicenter RCT design with recruitment targets able to achieve the power needed to make sound comparisons for our primary and important secondary outcomes in a sample which is representative for the continuum of HFpEF. To enhance external validity, the eligibility criteria for our population are broad and our recruitment strategy targets people from the age of 30 via different clinical units of (university) hospitals as well as the overall Flemish population. But at the same time the PRIORITY trial applies a thorough group allocation based on the latest guidelines combining echocardiography in rest and during exercise with biochemical parameters (NT-proBNP) to differentiate between HFpEF stages A, B and C. This recruitment strategy will enable us to evaluate the implementation potential in the real world and the overall effectiveness of our intervention.

Third, the design with the 2-year follow-up measurements is unique and will enable a high level of evidence of the longer-term effects on CV health, quality of life and physical activity behaviour of this hybrid strategy of providing person-tailored exercise therapy.

Fourth, PRIORITY has as its core concept the large-scale collection of sensed physical activity data (and patient input) via the sports watches and chest strap which allows us to better calculate internal training load during a 1-year period. In combination with the comprehensive phenotyping and behavioural data of our patients, we will develop predictive models by machine learning, to identify patients who are most likely to adopt a physically active lifestyle, increase their physical fitness or experience health benefits. This will enable the development of better-personalized exercise interventions and/or implementation strategies in the future.

Finally, implementation of effective lifestyle measures for the primary prevention of HF in patients at high risk and long-term secondary prevention of HF stage C remains suboptimal. Moreover, important socio-economic differences regarding risk factor control have been reported (49). The health economic analysis within PRIORITY will provide valuable insight into cost-effectiveness and quality-adjusted life years. This highly needed evidence could further guide the healthcare system on deciding which interventions are ready for reimbursement to maximize a society’s health gain.

This trial also has limitations that must be underlined. First, there is a risk for selection bias with individuals volunteering to participate being potentially more motivated to initiate exercise therapy. From eligible patients being referred for participation by their physician, the reason for non-participation will be recorded. Furthermore, the written exercise prescription given to the usual care comparator group is already more tailored than just the advice to be physically active and might encourage some patients to start exercising regularly. However, given the class IA recommendation we felt that providing no advice is unethical in this group. Finally, during the 2-year FU new medications might be initiated which could impact outcome of the participants. Though, given the randomized design, we anticipate that this will be equally distributed among the two trial arms. Further, medication and its change will be documented during the 2-year period.

## Conclusion

The PRIORITY trial aims to bridge the gap between guideline recommendations and the implementation into clinical practice by evaluating the effectiveness of a hybrid strategy for delivering personalized exercise therapy to the rapidly expanding population of patients with HFpEF stage A–C. The PRIORITY trial aims to provide the scientific evidence to support the use of remotely guided exercise therapy as an imperative preventive cost-effective treatment in the HFpEF continuum. The trial will focus on the prevention of progression of asymptomatic diastolic dysfunction towards symptomatic HFpEF (=primary prevention) and delaying progression of symptomatic HFpEF (=secondary prevention). This document provides a detailed description of the design, methodology and protocol of the PRIORITY trial. If the results of this trial are positive, this strategy of implementation of personalized exercise therapy can be easily extended to other patient populations with chronic diseases for whom exercise, and the adoption of a physically active heart healthy lifestyle are a core component in their disease management.

## Ethics statement

The studies involving human participants were reviewed and approved by Ethical Commission Research UZ/KU Leuven; Ethical Commission Research—Jessa Hospital; Ethicial Commission University Hospital Antwerp; Ethical Commission Research University Hasselt. The patients/participants provided their written informed consent to participate in this study.
