# Supplementary material for: Personalized remotely guided preventive exercise therapy for a healthy heart (PRIORITY): protocol for an assessor-blinded, multicenter randomized controlled trial
Source: Front Cardiovasc Med. 2023 Jun 29;10:1194693. doi: 10.3389/fcvm.2023.1194693 (PMC10339344; doi:10.3389/fcvm.2023.1194693)
Supplement: Supplementary file 1 [file Datasheet1.docx]

Supplementary File 1

Description of the intervention using the template for intervention description and replication (TIDieR) Checklist

| **Why?** In current practice, structured exercise therapy is mostly provided within a secondary prevention program for patients with heart failure with reduced ejection fraction. Presently, no comprehensive preventive care program exists that offers structured exercises for patients in the early stages of heart failure, when cardiovascular risk factors are present, or patients with heart failure with preserved ejection fraction. However, cardiac remodelling and dysfunction might still be reversible or even preventable. PRIORITY aims to investigate the use of remotely guided exercise therapy as a preventive clinical and cost-effective treatment in patients along the Heart Failure with preserved Ejection Fraction (HFpEF) continuum. |
| --- |
| **What materials? After completion of the baseline measurements,** all participants receive a personalized exercise prescription, generated by the EXercise Prescription in Everyday practice & Rehabilitative Training (EXPERT) tool^1^, and provided by a physiotherapist or movement scientist. The EXPERT tool automatically generates a tailored exercise prescription according to the characteristics (medication and devices, cardiovascular risk factors, cardiovascular diseases, presence of co-morbidities and the results of cardiopulmonary exercise test) of each patient, thus integrating the exercise prescriptions for different cardiovascular diseases and risk factors within the same patient, all based on ESC/EAPC recommendations, evidence, and expert opinions.^1^ Participants allocated to the PRIORITY group will also receive a sports watch (Garmin Forerunner 45s), a chest strap and resistance bands. Furthermore, patients with HFpEF stage C will receive a home ergometer (FitBike Ride 5) for at-home use. During the supervised training sessions in the center, various materials, which are also available to the patient at home, will be used (i.e. resistance bands, free weights, sports mats, stairs, cycle ergometers, and treadmils). Our website, [[www.inspanningstherapie.be](http://www.inspanningstherapie.be)](http://www.inspanningstherapie.be), will be used to provide online workouts for home-based training sessions, tailored to the individual. This website is composed of two parts. Part one is accessible to all visitors and provides visitors with information on the aim of the study, inclusion criteria, study procedures and contact details; the second part is only accessible to patients (and only Dutch language) who are randomized to the PRIORITY intervention but will be made accessible to the larger public after completion of the trial. This second part of the website offers work outs, single exercises for practice and FAQ. A snap shot of this can be seen below. As can be appreciated, patients can select the exercises/work outs which are recommended by the supervisors (i.e. using the numbers as provided by the researchers) but can also select exercises and work outs (using the filter) based on their expertise (low level of coordination, moderate, high, body parts, attributes- (elastic bands, weights, own body weight, no attributes), intensity (low, moderate, high) and position (standing, sitting, floor exercises).  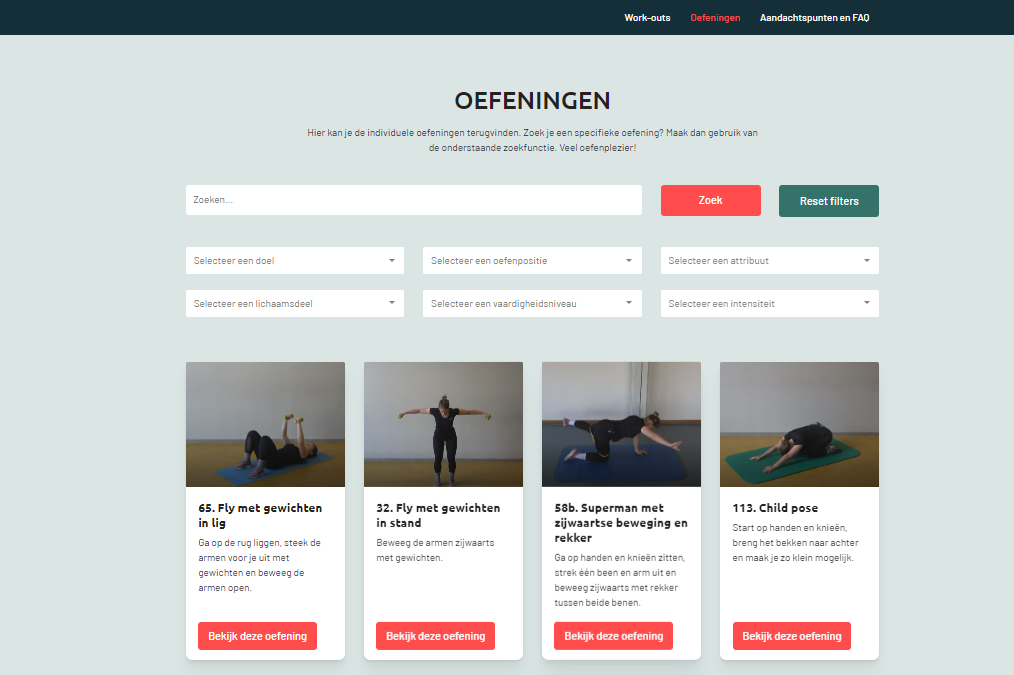  To objectively evaluate levels of physical activity, a research-based accelerometer (ActiGraph GT9X Link) will be given to both groups at baseline, at 4 months, 1-year and 2-year follow-up. |
| **What procedures?**  **PERSONALIZED EXERCISE PROGRAM (TRAINING GROUP).**  The intervention for the training group consists of four main components:   1. **Personalized exercise prescription:** This prescription will be generated by the EXPERT tool, a digital decision support system for optimized exercise prescription, endorsed by the European Association of Preventive Cardiology. FITT principles (frequency, intensity, type, time) and its implementation into daily practice will be discussed with the participant during the first visit. 2. **Supervised training sessions:** The supervised exercise program will consist of 18 exercise sessions during the first year. The number of supervised sessions will gradually decrease over time to encourage self-management and empowerment. This decrease will be implemented as follows: month 1 (weekly supervised session), month 2-4 (one supervised session every 2 weeks), month 5-12 (one supervised session per month). Each session consists of:    1. **Warm-up** (~ 5 min): Low intensity exercises.    2. **Aerobic training** (~ 30 min): Moderate and/or high intensity training (dependent on personalized exercise prescription) will be provided with a cycle ergometer / treadmill/stair walking. Furthermore, high-intensity interval training can be added in patients with HFpEF stage A and B, based on personal preferences of the patient. Exercise intensity will be prescribed as a heart rate training range based on the ventilatory thresholds (VT) defined during the baseline cardiopulmonary exercise test and adjusted based on the results of the cardiopulmonary exercise test at 4 months. To assess the first VT we use the nadir of the VE/VO2 to work rate (WR) relationship (i.e. the lowest point in the curve before VE/VO2 starts to increase) and the V-slope method in which the slope of the linear relation between VO2 and VCO2 increases (i.e. the increase in VCO2 becomes faster than the VO2 increase).^2^ VT2 corresponds to the nadir of the VE/VCO2 to WR relationship (i.e. the lowest point in the curve before VE/VCO2 starts to increase) and the VE/VCO2-slope method in which the slope of the linear relation between VE and VCO2 increases (i.e. the increase in VE becomes faster than the VCO2 increase).^2^These thresholds are then extrapolated to the corresponding HR to determine the ‘exercise training zones’. Training programs for the majority of our study population starts at a low-intensity (i.e. at an HR just below VT1) to acquainting them with exercise training (during the first week) and then evolves to moderate-intensity (at an HR between VT1 and VT2). From month 4 on, patients that show interest in high-intense training are prescribed HIIT with the high intensity bouts at an HR above VT2. Considerable flexibility with regard to the prescription of the high-intensity interval training will be adopted to allow for individual participant responses. For participants with HFpEF stage C, interval training at moderate intensity is provided and will be largely guided by the clinical signs of dyspnea. Perceived exertion will be rated by the BORG scale in which we aim for a score of 12-14 for moderate intensity and 15-16 when performing high intensity interval training. Heart rate will be monitored during each of the training sessions by means of the chest strap to evaluate the intensity. Training loads will be adjusted to the patient’s response as they progress through the exercise sessions, based on heart rate and subjective ratings.    3. **Dynamic strength training** (~ 30 min): Strength exercises with use of own body weight, resistance bands and free weights will be provided (e.g., squat, lunges, biceps curl…).. Number of sets and repetitions will be based on to the training goal: adaptation (2x15), hypertrophy (3x12) or endurance (2x20). Each strength session consists of approximately 8 exercises, targeting the large muscle groups, though personalized to the abilities of the patient. Load of resistance bands (i.e. color) is increased from the moment patients are able to comfortably perform the number of repetitions during three consecutive sessions Balance and coordination are part of every exercise session.    4. **Cooldown** (~ 5 min): Stretching exercises (quadriceps, hamstrings, calf muscles...). 3. **Home based training sessions:** Home-based training sessions will be prescribed by the treating physiotherapist or movement scientist, based on the individual level of the participant. Prescribed exercises consist of aerobic training and dynamic strength training which resemble the supervised sessions. Online workouts for the dynamic strength training will be provided at our website: [www.inspanningstherapie.be](http://www.inspanningstherapie.be). Frequency of the training sessions and total volume will depend on the personalized exercise prescription with a minimal of 3 sessions per week. 4. **Feedback and advice:** Patients will receive feedback on their performed training sessions at home (i.e. uploaded data on GarminConnect platform) during the supervised training sessions. Adherence will be evaluated, based on agreement between prescribed frequency, intensity and volume of exercise.. After 1 year of training, patients will exercise independently and will have a one-on-one physical activity counselling session at 3 specified timepoints over the following year.   **COMPARATOR GROUP**  Participants in the comparator group will receive a personalized exercise prescription and the advice to exercise independently. |
| **Who provided?** The intervention will be administered by a physiotherapist or movement scientist who will educate and provide aerobic endurance training, dynamic strength training, coordination and flexibility exercise and home exercises per the trial protocol. |

Supplementary File 2

*Rest transthoracic echocardiography*

All participants refrain from alcohol or caffeine consumption, vigorous exercise, and smoking. To ensure a stable condition, echocardiography will be performed after the subject has been in the supine position for at least 15 minutes.

*Imaging protocol*

Two- and three-dimensional TTE will be performed using a Vivid E95 or E9 ultrasound system (GE Vingmed, Horten, Norway) interfaced with a 1.4 – 5.2 MHz phased-array transducer. With the subjects in partial left decubitus, the observer will obtain images along the parasternal long- and short-axes and along the apical 4-, 3- and 2-chamber views, and the subcostal view, with a simultaneous electrocardiogram signal. All images will include at least 5 cardiac cycles and will be digitally stored for offline analysis using EchoPAC (version 204; GE Healthcare, Horten, Norway).

All measurements will be made and analyzed following international guidelines^1,2^. Cardiac morphology for both ventricles will be assessed, including end-diastolic volume and end-systolic volume in 2D (biplane disk summation) and 3D, rendering left ventricular (LV) ejection fraction (LVEF). LV internal diameter (LVID), interventricular septum (IVS) and posterior wall thickness (PWT) will be measured from a 2D parasternal long-axis image at end-diastole. End-diastolic LV dimensions will be used to calculate LV Mass (LVM) using the Linear Cube formula: 1.04*((LV Internal Diastolic Diameter + Interventricular Septum + LV Posterior Wall Thickness)³ - (LV Internal Diastolic Diameter³))*0.8+0.6. Left atrial (LA) volume will be assessed by the biplane area-length method from apical four- and two-chamber views. LVM and LA volumes will be indexed to body surface area (BSA), calculated as: 0.007184 x (body length (in cm)^0.725) x (body weight (in kg)^0.425). The left ventricular outflow tract (LVOT) will be measured from the parasternal long-axis view at baseline. The diastolic function will be assessed using established Doppler and tissue-Doppler parameters such as the E wave velocity, the A wave velocity, the E/A ratio, septal, lateral and averaged E’, E/E’, tricuspid regurgitation flow velocity, and the S-D-A waves at the pulmonary veins. To assess the LV systolic function, besides LVEF, the global longitudinal strain (GLS) will be calculated according to the current guidelines (>50 fps).

*Exercise echocardiography combined with CPET (CPET-echo)*

*Exercise protocol*

Exercise will be performed on a semi-supine bicycle ergometer (Ergoline GmbH, Bitz, Germany) with a continuous ramp protocol based on the previous CPET (i.e. upright bicycle) dividing the maximum workload by 10 and rounding down to obtain the starting workload and the increase per minute (e.g. after a maximum load of 137W at the previous CPET, a protocol of 10+10W will be selected). Participants are asked to cycle with a frequency of 60-65 rotations/minute. Images will be acquired at rest, at low intensity (HR between 90-100 beats per minute, before fusion of E and A waves, or at RER between 0.85-0.9 when chronotropic incompetence is present), and at peak exercise (RER 1.05). Loop registration of at least 10 beats will be made to overcome the expected decrease in acoustic quality caused by hyperventilation. At one year of follow-up, the CPET-echo study will follow the same imaging and ramp protocol. The power output at low-intensity exercise will be identical to the low-intensity workload during the baseline CPET-echo at the time of inclusion. In contrast, the power output of the peak exercise stage will be determined based on the criterion of achieving RER 1.05.

*Exercise imaging protocol*

The standardized imaging protocol during exercise includes an apical four-chamber image, apical two-chamber image, apical three-chamber image, four-chamber Tissue Doppler Image (TDI), septal pulsed wave (PW) TDI, lateral PW TDI, PW of mitral inflow, PW of the LVOT, an image of the right ventricle and a continuous wave signal of tricuspid regurgitation jet. Agitated colloids (Gelofusine 4%, Braun, Melsungen, Germany) will be used in all participants through injection in a left antecubital vein to optimally delineate the tricuspid regurgitation envelope, as previously described by our team^3^. An image of the dimension and collapsibility of the inferior vena cava will be assessed to estimate right atrial pressure (RAP) at each stage. B-lines will be assessed at rest and peak exercise in four lung zones (basal lateral and upper anterior at each side). Each lung zone will be evaluated separately and classified as positive in the presence of three or more B-lines. A final score will be derived by summation of the scores for all four lung zones (1 field = mild; 2 or 3 fields = moderate; 4 fields = severe). Cardiac output (CO) will be evaluated using the velocity-time integral of the LVOT via PW Doppler, heart rate (HR), and LVOT diameter. The latter will be obtained from a parasternal long-axis view during previous rest transthoracic echocardiography in the left lateral decubitus position. Measures of diastolic function will only be derived before E/A fusion and will include mitral inflow early (E) and late (A) flow velocities by PW Doppler, and early diastolic velocity (e’) at the septal and lateral annulus by Tissue Doppler Imaging. These measurements will derive E/e’ (average of septal and lateral values) and systolic pulmonary artery pressure (sPAP) will be estimated from a colloid-enhanced TR velocity signal without adding RAP. Mean PAP (mPAP) will then be calculated by the Chemla formula as sPAP*0.61+2. All analysis will be performed offline at the core lab of Leuven using EchoPAC software (version 204, GE Vingmed) in accordance with contemporary international guidelines^4,5^.

References:

1. Hansen D, Dendale P, Coninx K*, et al.* The European Association of Preventive Cardiology Exercise Prescription in Everyday Practice and Rehabilitative Training (EXPERT) tool: A digital training and decision support system for optimized exercise prescription in cardiovascular disease. Concept, definitions and construction methodology. *Eur J Prev Cardiol* 2017;**24**:1017-1031. doi: 10.1177/2047487317702042

2. Hansen D, Abreu A, Ambrosetti M*, et al.* Exercise intensity assessment and prescription in cardiovascular rehabilitation and beyond: why and how: a position statement from the Secondary Prevention and Rehabilitation Section of the European Association of Preventive Cardiology. *Eur J Prev Cardiol* 2022;**29**:230-245. doi: 10.1093/eurjpc/zwab007

3. Claessen G, La Gerche A, Voigt JU*, et al.* Accuracy of Echocardiography to Evaluate Pulmonary Vascular and RV Function During Exercise. *JACC Cardiovasc Imaging* 2016;**9**:532-543. doi: 10.1016/j.jcmg.2015.06.018

4. Lang RM, Badano LP, Mor-Avi V*, et al.* Recommendations for cardiac chamber quantification by echocardiography in adults: an update from the American Society of Echocardiography and the European Association of Cardiovascular Imaging. *Eur Heart J Cardiovasc Imaging* 2015;**16**:233-270. doi: 10.1093/ehjci/jev014

5. Nagueh SF, Smiseth OA, Appleton CP*, et al.* Recommendations for the Evaluation of Left Ventricular Diastolic Function by Echocardiography: An Update from the American Society of Echocardiography and the European Association of Cardiovascular Imaging. *Eur Heart J Cardiovasc Imaging* 2016;**17**:1321-1360. doi: 10.1093/ehjci/jew082

4. Voigt JU, Pedrizzetti G, Lysyansky P, Marwick TH, Houle H, Baumann R, Pedri S, Ito Y, Abe Y, Metz S, Song JH, Hamilton J, Sengupta PP, Kolias TJ, d'Hooge J, Aurigemma GP, Thomas JD, Badano LP. Definitions for a common standard for 2D speckle tracking echocardiography: consensus document of the EACVI/ASE/Industry Task Force to standardize deformation imaging. Eur Heart J Cardiovasc Imaging. 2015 Jan;16(1):1-11. doi: 10.1093/ehjci/jeu184. Epub 2014 Dec 18. PMID: 25525063.
